# Breast 3 T-MR imaging: indication for stereotactic vacuum-assisted breast biopsy

**DOI:** 10.1186/2193-1801-3-481

**Published:** 2014-08-28

**Authors:** Nobuko Yamamoto, Takeshi Yoshizako, Kazuaki Yoshikawa, Masayuki Itakura, Riruke Maruyama, Hajime Kitagaki

**Affiliations:** Department of Radiology, Shimane University Faculty of Medicine, 89-1 Enya, Izumo, Shimane, 693-8501 Japan; Department of Surgery, Shimane University Faculty of Medicine, 89-1 Enya, Izumo, Shimane, 693-8501 Japan; Department of Pathology, Shimane University Faculty of Medicine, 89-1 Enya, Izumo, Shimane, 693-8501 Japan

**Keywords:** 3 T-MR imaging, Breast cancer, Microcalcifications, Mammography, Stereotactic vacuum-assisted breast biopsy

## Abstract

**Purpose:**

The purpose of this study was to assess indications for stereotactic vacuum-assisted breast biopsy (SVAB) evaluated by breast 3 T-magnetic resonance (3 T-MR) imaging in patients showing suspicious microcalcifications on mammography and negative ultrasound (US) findings.

**Methods and materials:**

Fifty-five patients with 55 breast lesions showing suspicious microcalcifications on mammography and negative US findings underwent preoperative 3 T-MR examination including dynamic MR imaging. All patients underwent SVAB within 1 month of MR imaging. The pathological diagnosis of each breast lesion was made by examining tissues obtained by SVAB or radical/partial mastectomy.

3 T-MR imaging findings were evaluated by using the American College of Radiology Breast Imaging Reporting and Data System Atlas (BI-RADS-MRI) and then were correlated with the histopathological findings. When BI-RADS 4 and 5 MR imaging lesions were assumed to be malignant, the usefulness of 3 T-MR imaging was evaluated for diagnosis of impalpable breast lesions by SVAB among lesions with microcalcification detected by mammography and negative US findings.

**Results:**

There were 21 malignant lesions, including 5 invasive ductal carcinomas, 16 lesions of ductal carcinoma in situ (DCIS). The sensitivity, specificity, positive predictive value, negative predictive value, and accuracy of 3 T-MR imaging for deciding the indications for SVAB was 90.5%, 97.1%, 95.0%, 94.3%, and 94.5%, respectively. The one-false negative case was a DCIS with small enhancing lesions (0.5 mm). The one false-positive case was ductal adenoma with a linear ductal pattern of enhancement.

**Conclusions:**

3 T-MR imaging may be useful for deciding the indications for SVAB in patients who have breast lesions with microcalcification that are impalpable and are detected by mammography and negative US findings. However, our findings should be considered preliminary and further prospective investigation is required.

## Introduction

Percutaneous imaging-guided breast biopsy is widely used to evaluate predominantly impalpable breast lesions. There has been steady development of percutaneous biopsy techniques and stereotactic vacuum-assisted breast biopsy (SVAB) has been established as a reliable method for the diagnosis of impalpable lesions with microcalcification detected by mammography (Kikuchi et al. 2007; Nakamura et al. 2010). The high accuracy of SVAB diagnosis has led to a steady decline in the performance of diagnostic open surgical biopsy (O'Flynn et al. 2010). SVAB is much less invasive than conventional open biopsy. However, it still involves physical and mental burdens for the patient, so it is important to avoid unnecessary procedures.

Breast magnetic resonance (MR) imaging has increasingly been performed over the past 10 years because of its well-documented high sensitivity for detecting breast cancer, especially occult tumors missed by conventional imaging modalities (Kuhl et al. 2007; Pediconi et al. 2007; Lehman et al. 2007; Berg et al. 2004). Since malignant tumors typically exhibit increased vascularity, an early remarkable enhancement and some specific pattern of contrast enhancement, an essential part of many breast MR studies is T1-weighted dynamic contrast-enhanced imaging.

In recent years, MR scanners with stronger magnetic fields (such as 3 T scanners) and thus a higher signal-to-noise ratio have become more widely available and have opened up new horizons for contrast-enhanced breast MR imaging (Elsamaloty et al. 2009; Kuhl et al. 2006; Rahbar et al. 2013; Lourenco et al. 2014).

The purpose of this study was to assess indications for stereotactic vacuum-assisted breast biopsy (SVAB) evaluated by breast 3 T-MR imaging in patients showing suspicious microcalcifications on mammography and negative US findings.

## Methods and materials

### Patients

Our Institutional Review Board approved this retrospective study and the need to obtain informed consent was waived. The inclusion criteria were women undergoing screening of lesions with microcalcification classified as Breast Imaging Reporting and Data System (BI-RADS) (American College of Radiology 2003) categories 3, 4, or 5 without a detectable mass on mammography or US. A retrospective review was performed of all patients who underwent 3 T-MR imaging for evaluation of calcified breast lesions from January 2010 to June 2012 and who had pathologic confirmation of the diagnosis. Pathological diagnosis of the breast lesions was performed by examining tissues obtained by SVAB or radical/partial mastectomy. The general exclusion criteria for MR imaging (claustrophobia, pregnancy, pacemaker, etc.) were applied.

A total of 57 patients with microcalcifications considered to be BI-RADS category 3 and over, detected on mammography, were initially recruited at our institution. However two patients were excluded because of positive US findings. Moreover, one person with category 3 was performed a SVAB on the hope of the patient. As a consequence, this study included 55 women with calcified breast lesions who underwent 3 T-MR breast imaging. All patients subsequently underwent SVAB within 1 month of MR imaging. The mean age was 53 years (range: 31–82 years). MR imaging of both breasts was performed in all patients, so imaging data for 110 breasts were obtained.

### Imaging protocols

#### Mammography and interpretation

Bilateral digital mammography was performed using a LoRad M-IV (Lorad/Hologic, Danbury, Conn., USA). Images of both breasts were obtained in the routine craniocaudal and mediolateral oblique views along with spot-magnification views of the areas with microcalcification. Digital mammograms were independently read by two experienced radiologists (N. Y., K. Y.) using the BI-RADS assessment categories (American College of Radiology 2003). If the two readers differed in their assignment of BI-RADS categories, they reached a consensus by discussion. Microcalcification was classified according to the BI-RADS description of mammography features, including assessment of the morphology (punctate, amorphous, pleomorphic, or linear) and the distribution (diffuse, regional, clustered, segmental, or linear) (American College of Radiology 2003).

### Breast US and interpretation

Based on the clinical and mammography findings, bilateral whole breast US was performed before MR imaging and SVAB. US was done with a linear array broadband transducer at a central frequency of 10–12 MHz (EUB-7500; Hitachi Medical Corporation, Tokyo, Japan), and the findings were interpreted by a single experienced radiologist (N. Y.). Based on the US findings, lesions that required US-guided core needle breast biopsy in the judgment of a single experienced surgeon (M. I.) were excluded from this study.

### Breast MR imaging and interpretation

MR imaging was performed using a 3.0 T system (Signa HDx, GE Healthcare, Milwaukee, WI, USA). A body coil was employed for transmission, and a double breast coil (eight-channel breast array coil) was used for receiver. MR examinations have not considered the menstrual cycle.

Before administration of contrast medium, sagittal and coronal fat-suppressed T2-weighted images (TR/TE, 5,000/80; field of view, 20 cm; matrix, 256 × 224; slice thickness, 4 mm; acquisition time, 135 seconds) were obtained of the breast with microcalcification.

Then dynamic MR imaging was performed using a volume imaging for breast assessment (VIBRANT) sequence with parallel acquisition. VIBRANT sequences were acquired before and four times after injection of a bolus of gadodiamide (0.1 mmol/kg; Omni scan, Daiichi Sankyo Tokyo Japan) at a rate of 2 mL/s (followed by flushing with 20 mL of saline) using an automatic injector. Both breasts were examined in the axial plane using the first-, second-, third-, and fourth-phase dynamic images acquired at 1.5, 3, 4.5, and 6 minutes after contrast injection, respectively. The parameters for dynamic MR imaging were as follows: TR/TE, 5.8/2.1; flip angle, 10°; field of view, 28 cm; matrix, 320 × 320; receiver bandwidth, 260 Hz per pixel; interpolated slice thickness, 1.4 mm; partitions, 128; and acquisition time, 90 seconds.

Image analysis was performed on a GE workstation. Two radiologists (N. Y. had 11 years and T. Y. had 13 years of experience in breast MR imaging), who made consensus decisions about the diagnosis, evaluated the contrast-enhanced MR images retrospectively. They were aware of the mammography findings, and assigned each breast to a category on a patient-to-patient basis. For diagnosis, the BI-RADS-MRI classification proposed by the American College of Radiology (Molleran & Mahoney 2013) was used. Early enhancement patterns were evaluated on the first- and second-phase dynamic images, while delayed enhancement was assessed on the third-phase images. Early enhancement patterns were based on dynamic signal intensity-time curves. Two radiologists (N. Y., T. Y.) obtained dynamic signal intensity-time curves by placing a region of interest in the area of maximal enhancement within the microcalcification lesions. Lesions were categorized using the flowcharts and interpretation method of Tozaki et al. (Tozaki & Fukuda 2006; Tozaki & Fukuma 2009) and Akita et al. (Akita et al. 2009) (Table 1).

**Table 1:** **Categorization of breast lesions on contrast**-**enhanced MRI**

BI- RADS	Appearance on contrast- enhanced MR imaging
**Mass lesion**	
Category 5	Spiculated margin Irregular lesion: rapid washout pattern and rim enhancement
Category 4b	Irregular lesion Smooth margin: washout pattern
Category 4a	Smooth margin: nonwashout and initial rapid rise
Category 3	Smooth margin: neither washout nor initial rapid rise
**Non-** **mass lesion**	
Category 5	Segmental distribution and clustered ring enhancement
Category 4b	Segmental distribution, Branching ductal pattern, Clustered ring enhancement, Clumped architecture
Category 4a	Linear ductal pattern
Category 3	Not showing the characteristics of category 4 or 5
**Focus(<** **5 mm)**	
Category 4a	Rapid washout pattern
Category 3	Without rapid washout pattern
Category 1, 2	No abnormal enhancement, bilaterally symmetrical enhancement

### SVAB protocol and management

First, the patient was positioned prone on a digital stereotactic table (LoRad DSM; Lorad/Hologic, Danbury, Conn., USA). Then SVAB was performed by one radiologist or one surgeon using a vacuum-assisted biopsy device with a 7 or 8-gauge probe (Mammotome; Ethicon EndoSurgery, Cincinnati, Ohio, USA). After SVAB, digital radiography of the biopsy specimen was routinely performed to check whether the tissue containing microcalcification had been collected. If malignancy was confirmed by pathological examination of the SVAB specimen, the surgeon proceeded to definitive surgical intervention. If SVAB revealed a high-risk lesion, such as atypical ductal hyperplasia (ADH), the surgeon performed excision biopsy. If a benign lesion was detected by pathological examination, the patient was scheduled for repeat mammography of the ipsilateral breast after 6 months and annual screening mammography was recommended thereafter. In this study, a diagnosis of “benign” was defined as no malignancy on SVAB and no change of the microcalcification on follow-up mammography for one year.

### Histopathological examination

The histopathological diagnosis was determined by a single experienced pathologist (R. M.). The reference standard was serial 5-mm slices of the surgical specimens. The maximum diameter of the malignant lesions was estimated by one pathologist by tumor mapping based on the results of microscopic examination.

### Data analysis

In the BI-RADS classification, categories 4 and 5 are considered to be malignant. In the MR classification, categories 4 and 5 were also considered to be malignant. We calculated the sensitivity, specificity, positive predictive value (PPV), negative predictive value (NPV), and accuracy of mammography alone and mammography plus MR imaging for diagnosing microcalcification as benign or malignant using 2 × 2 contingency tables. Fisher’s exact test and the Mann–Whitney U-test were employed to examine statistical significance. All statistical analyses were performed using SPSS software (version 22, SPSS) and P < 0.05 was considered statistically significant.

## Results

### SVAB and histopathology

SVAB was performed successfully in all 55 patients without any complications. Digital radiography confirmed that all specimens contained microcalcification. Examination of the biopsy specimens revealed that 21 patients (38.2%) had carcinoma and 34 patients (61.8%) had benign disease (Table 2). In the patients with malignant lesions, surgical excision was performed. The final histopathological diagnosis was invasive ductal carcinoma in five patients, DCIS in 16 patients. The DCIS lesions were classified as low, intermediate, and high grade in 3, 7, and 6 cases, respectively. The maximum diameter of the 16 DCIS lesions ranged from 0.5 to 110 mm (mean: 38.2 mm), while the maximum diameter of the 5 invasive ductal carcinomas ranged from 9 to 30 mm (mean: 17.1 mm). For all malignant lesions, the mean maximum diameter was 21.5 mm. The 34 patients with benign disease had mastopathy (n = 7), calcification (17), ductal adenoma (n = 1), fibrocystic change and sclerosing adenosis (n = 1), intraductal papilloma (n = 1), papillomatosis (n = 1), atypical ductal hyperplasia (n = 2), and other benign diseases (n = 4) according to histopathologic examination. These patients with benign lesions were advised to undergo follow-up imaging according to our institutional protocol, and all 34 complied. The duration of follow-up ranged from 370 to 860 days. There was no evidence of false-negative diagnosis because no progression of microcalcification at the site of SVAB was revealed by follow-up mammography.

**Table 2:** **Stereotactic vacuum**-**assisted breast biopsy** (**SVAB**) **analysis and Histopathology**

Benign	34
Ductal adenoma	1
Intraductal papilloma	1
Sclerosing adenosis	1
Epitheliosis (duct papillomatosis)	1
Mastopathy	**7**
Calcification	17
ADH (atypical ductal hyperplasia)	2
No malignancy	4
**Malignancy**	21
IDC (papillotubular carcinoma)	5
DCIS (high grade)	6
DCIS (intermediate grade)	7
DCIS (low grade)	3
**Total**	**55**

### Mammography findings

When assessment of the mammography findings was based on the BI-RADS categories, one of the 55 lesions (1.8%) was assigned to category 3, while 42 lesions (76.4%) were category 4 and 12 lesions (21.8%) were category 5 (Table 3). The PPV for malignancy in categories 3, 4, and 5 was 0% (0/1), 26.2% (11/42), and 83% (10/12), respectively.

**Table 3:** **Morphology and distribution of microcalcifications on mammography**

Calcification	BI-RADS category			Total	Histopathology	
	3	4	5		Benign	Malignancy
**Morphology**						
Punctate	1	-	-	1	1	-
Amorphous	-	10	7	17	10	7
Pleomorphic	-	32	5	37	23	14
Linear	-	-	-	-	-	-
**Distribution**						
Diffuse	-	1	-	1	-	1
Regional	-	1	-	1	1	-
Clustered	-	28	6	34	22	12
Segmental	1	11	6	18	10	8
Linear	-	1	-	1	1	-
**Total**	1	42	12	55	34	21
Benign	1	31	2	34		
Malignancy	-	11	10	21		

With regard to morphology, the one lesion with punctate microcalcification was benign, while 41.2% of lesions with amorphous microcalcification (7/17) and 37.8% of those with pleomorphic microcalcification (14/37) were malignant. With respect to the distribution of calcification, 100% of the lesions showing diffuse microcalcification (1/1), 0% of those with regional microcalcifications (0/1), 35.3% of lesions with clustered microcalcification (12/34), and 44.4% of lesions showing segmental microcalcification (8/18) were malignant.

### Breast MR imaging findings

Fifty-five patients with impalpable 55 breast lesions showing suspicious microcalcifications on mammography and negative US findings underwent preoperative 3 T-MR examination, including dynamic MR imaging. There were no women who could not get scanned due to renal problems/claustrophobia etc. Moreover, second another lesion were not identified at MR imaging in this patient group.

**Figure 1:**
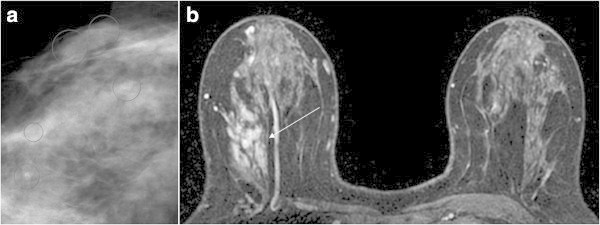
**Ductal carcinoma in situ** **(DCIS)** **with calcifications in the right breast of a 50**-**year**-**old woman.** **(a)** Right craniocaudal mammogram with spot compression magnification demonstrates non mass-like segmental amorphous microcalcifications classified as BI-RADS category 4 (circle). **(b)** Axial contrast-enhanced T1-weighted fat-suppressed MR images of bilateral breasts is showing non mass-like segmental distribution and clustered ring enhancement in the area of microcalcifications (arrow). This finding was classified as BI-RADS category 5.

**Figure 2:**
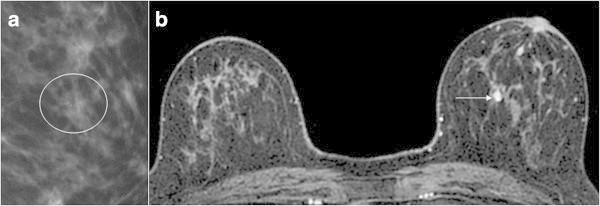
**Secretory form calcifications in the left breast of a 62**-**year**-**old woman.** **(a)** Left mediolateral mammogram with spot compression magnification demonstrates non mass-like clustered amorphous microcalcifications classified as BI-RADS category 4 (circle). **(b)** Axial contrast-enhanced T1-weighted fat-suppressed MR images of bilateral breasts is showing non mass-like focus enhancement in the area of microcalcifications (arrow). This finding was classified as BI-RADS category 3.

Based on MR imaging findings, 21 lesions were in categories 1 or 2, 14 lesions were in category 3, one lesion was in category 4a, 17 lesions were in category 4b, and two lesions were in category 5 (Table 4, Figures 1 and 2). Category 1 (no abnormal enhancement) and category 2 (bilateral symmetrical enhancement) accounted for 20 benign lesions and one malignant lesion (high-grade DCIS with a diameter of 0.5 mm). In category 3, there were 13 benign lesions and one malignant lesion (low grade DCIS). The one lesion in category 4a was benign (ductal adenoma). Category 4b had no benign lesions and all 17 lesions in this category were malignant. Both of the two lesions in category 5 were also malignant.

**Table 4:** **Categorization and case number of breast lesions on contrast-** **enhanced MRI**

BI- RADS category	Appearance on contrast- enhanced MR imaging	Sub- total	Benign	Malignancy
Mass lesion				
Category 5	Spiculated margin Irregular lesion: rapid washout pattern and rim enhancement	-	-	-
Category 4b	Irregular lesion Smooth margin: washout pattern	1	-	1
Category 4a	Smooth margin: nonwashout and initial rapid rise	-	-	-
Category 3	Smooth margin: neither washout nor initial rapid rise	-	-	-
Non-mass lesion				
Category 5	Segmental distribution and clustered ring enhancement	2	-	2
Category 4b	Segmental distribution, Branching ductal pattern, Clustered ring enhancement, Clumped architecture	16	-	16
Category 4a	Linear ductal pattern	1	1*	-
Category 3	Not showing the characteristics of category 4 or 5	6	6	-
Focus (<5 mm)				
Category 4a	Rapid washout pattern	-	-	-
Category 3	Without rapid washout pattern	8	7	1 (DCIS)
Category 1, 2	No abnormal enhancement, bilaterally symmetrical enhancement	21	20	1**
Total		55	34	21

*Ductal adenoma, **DCIS with a diameter of 0.5 mm

Among lesions classified as category 4 or higher, only one malignant lesion was a mass lesion, while one benign lesion and 18 malignant lesions were non mass lesions.

The detection rate of malignancy in category 3, category 4, and category 5 was 7.1% (1/14), 94.4% (17/18), and 100% (2/2), respectively. Malignant lesions were more frequent when the MR imaging diagnosis was positive (categories 4 or 5) than when MR imaging was negative (categories 1, 2, or 3) (Fisher’s exact test, P < 0.001). The number of benign and malignant lesions in each category was significantly different (Mann–Whitney U-test, P < 0.001).

If BI-RADS categories 4 and 5 were assumed to be malignant, for selecting lesions that required SVAB, 3 T-MR imaging for lesions with microcalcification had a sensitivity of 90.5%, specificity of 97.1%, PPV of 95.0, NPV of 94.3%, and accuracy of 94.5%.

## Discussion

Mammographically detected microcalcification is a frequent feature of breast tumors with early diagnosis, and is found in approximately 70% of minimal breast cancers and frequently detected in DCIS (Stomper & Margolin 1994). Stomper et al. (Stomper et al. 1989) reported that the presence of microcalcification on mammography was the only sign in 72% of clinically occult DCIS lesions. Because of the wider adoption of mammography, an increasing number of women with microcalcification on mammography are undergoing SVAB for more detailed examination. SVAB has some advantages for assessing microcalcification because it shows excellent sensitivity and specificity with a very low false-negative rate (Kettritz et al. 2004; Pfarl et al. 2002). However, approximately 75% of lesions that are detected, suspected, or indeterminate on mammography are found to be benign by biopsy (Kopans 1989), implying that many patients undergo biopsy unnecessarily because the indications for SVAB have not yet been fully established.

In the present study, when categories 4 and 5 of BI-RADS were assumed to be malignant, 3 T-MR imaging for lesions with microcalcification had a high sensitivity, specificity, PPV, NPV and accuracy for deciding the indication for SVAB. The one false negative lesion (high-grade DCIS) were less than 1.0 mm in diameter on pathology and was very small clustered pleomorphic microcalcification and bilaterally symmetrical enhancement. Also, there was no malignant disease around these lesions on excisional biopsy. The prognosis of such cases might be favorable, so frequent follow up would be one choice. Therefore, 3 T-MR imaging for lesions with microcalcification might have sufficiently high sensitivity, specificity, PPV, NPV, and accuracy to decide the indications for SVAB. 3 T-MR imaging may be useful to determine candidate lesions for SVAB after mammography detects microcalcification. When the candidate lesion shows abnormal enhancement along with calcification, it should be subjected to SVAB. If the lesion has no abnormal enhancement on MR imaging, follow up could be a good choice.

Of course, 3 T-MR imaging depends on the ability of radiologists to identify and accurately characterize breast lesions. At a higher field strength, improved detection can be achieved through better spatial resolution, more homogeneous fat suppression, and a higher contrast-to-noise ratio, while characterization of lesions may be improved through better spatial and/or temporal resolution. This should allow better visualization and characterization of enhancing lesions, which may improve the detection of breast cancer (Rahbar et al. 2013; Lourenco et al. 2014). Lourenco et al. (Lourenco et al. 2014) reported that the detection rate of cancer and the PPV of BI-RADS in breast screening were improved by 3 T-MR imaging compared with 1.5 T-MR imaging.

In our study, lesions were categorized using the interpretation flowcharts and interpretation method of Tozaki et al. (Tozaki & Fukuda 2006; Tozaki & Fukuma 2009) and Akita et al. (Akita et al. 2009). Tozaki et al. (Tozaki & Fukuda 2006) reported that the features of microcalcification with the highest PPV for carcinoma were a segmental distribution (100%), clustered ring enhancement (100%), and clumped internal architecture (88%). Using their interpretation model, they reported that the PPV for carcinoma was 94%. The results of our study were similar to their findings. One false-positive non-mass lesion (ductal adenoma) was found by MR imaging criteria in our study. This false-positive lesion (ductal adenoma) showed linear ductal enhancement. Tozaki et al. (Tozaki & Fukuda 2006) have suggested that linear enhancement might be stratified into two categories (linear nonspecific and linear ductal patterns) because the frequency of malignancy in these categories is different.

Akita et al. (Akita et al. 2009) reported that no malignancy was found in category 1 (no abnormal enhancement in both breasts) and category 2 (bilateral symmetrical enhancement), which may have contributed to the high specificity (100%) in their study. However, there was one false-negative case belonging to category 2 in our study. DCIS sometimes shows indistinct enhancement on MR imaging because of relatively poor or absent angiogenesis (Ghai et al. 2005). Moreover, the one false-negative lesion (high-grade DCIS) was less than 1.0 mm in size and thus were very small. Therefore, it should be remembered that very small calcified lesions (less than 1.0 mm) may be false negative on MR imaging.

This study had several limitations. First, it was a retrospective analysis. As a result, there is a difficulty in reproducibility of the result by various bias occurring, so prospective confirmation is required. Second, there was a relatively small study population. Third, the study was limited by a short follow-up period for benign lesions diagnosed by SVAB. Thus, a larger study and more outcome data are needed to confirm our results.

In conclusion, 3 T-MR imaging may be useful for deciding the indications for SVAB in patients who have breast lesions with microcalcification that are impalpable and are detected by mammography and negative US findings. However, our findings and conclusions should be considered preliminary and further prospective investigation is required.
